# Phosphorylation of P68 RNA Helicase by P38 MAP kinase contributes to colon cancer cells apoptosis induced by oxaliplatin

**DOI:** 10.1186/1471-2121-13-27

**Published:** 2012-10-31

**Authors:** Heena Dey, Zhi-Ren Liu

**Affiliations:** 1 Department of Biology, Georgia State University, Atlanta, GA 30303, USA

**Keywords:** P68 RNA helicase, Oxaliplatin, Phosphorylation, p38 MAP kinase, DEAD-box, Apoptosis

## Abstract

**Background:**

We previously demonstrated that p68 phosphorylation at threonine residues correlates with cancer cell apoptosis under the treatments of TNF-α and TRAIL (Yang, L. Mol Cancer Res Vol 3, pp 355–63 2005).

**Results:**

In this report, we characterized the role of p68 phosphorylation in apoptosis induction under the treatment of oxaliplatin in the colon cancer cells. Our data suggest that oxaliplatin treatment activates p38 MAP kinase, which subsequently phosphorylates p68 at T564 and/or T446. The phosphorylation of p68, at least partially, mediates the effects of the drug on apoptosis induction, as mutations at these two sites greatly reduce the cancer cell death.

**Conclusion:**

Our studies reveal an important molecular mechanism that mediates the effects of anti-cancer drug, providing a potential strategy for improving cancer treatment.

## Background

Oxaliplatin is a new generation of platinum derivatives that is currently used in the front line for the treatment of human colorectal cancer and other cancers [1]. The therapeutic effects of oxaliplatin may result from the DNA damage caused by the compound, which lead to cell cycle arrest and apoptosis [2,3]. The compound can induce DNA damage by the formation of cross-links between the two strands of DNA, leading to blockage of DNA replication and transcription [3-5]. The compound activates multiple signaling pathways in mediating apoptosis induction [6,7]. It is known that treatment of cancer cells with oxaliplatin results in the activation of p38 and/or JNK kinases, which subsequently target a number of downstream effector molecules leading to cell apoptosis. Although the mechanism underlying the tumor apoptosis induced by the drug has been intensively studied, the detailed mechanism, especially the cellular molecules that contribute to the effects of the drug, is not fully understood.

P38 is a stress-activated MAP kinase that is activated in response to many cellular stress induction signals, including oxidative stress and toxic chemicals [8,9]. Sustained activation of p38 MAP kinase is critical in mediating the effects of the stress signals in the induction of cell apoptosis [10,11]. A number of anti-cancer agents act via activation of p38, such as platinum compounds [12], etoposide [13], and taxol [14]. P38 Map kinase is activated by phosphorylation at Thr180 and Tyr182 residues in its conserved TGY motif [15,16]. The p38 MAP kinase targets a number of very important downstream proteins to exert its effects in apoptosis induction. It is reported that phosphorylation of p53 on Ser46 by p38 is essential for apoptosis induction by several anti-cancer drugs and virus [17,18]. Treatment of colon cancer cells with oxaliplatin leads to activation of p38 MAP kinase, which subsequently phosphorylates gamma-H2AX and securin. These phosphorylation events contribute to the cell apoptosis induced by the compound [19,20].

The nuclear p68 RNA helicase is a member of the DEAD box family of RNA helicase [21,22]. P68 RNA helicase plays a very important role in cell proliferation and early organ development and maturation [23]. The expression of the protein was shown to correlate with tumor progression and transformation [24]. We have previously reported that p68 RNA helicase is phosphorylated at multiple amino acid residues, including serine/threonine and tyrosine [25]. P68 was phosphorylated at tyrosine residue(s) in a number of different cancer cell lines but not in the corresponding normal cells/tissues. In response to growth factor PDGF-BB stimulation, p68 is phosphorylated at Y593 by c-Abl in HT-29 cells. Phosphorylation of p68 at Y593 promotes EMT via promoting β-catenin nuclear translocation [26]. P68 acquires a double tyrosine phosphorylation at Y593/Y595 in T98G glioblastoma cells. The double phosphorylation mediates resistance to TRAIL-induced apoptosis. Interestingly, when the cancer cells become apoptotic resistant, double tyrosine phosphorylations of p68 increases while threonine phosphorylation of p68 decreases indicating that p68 threonine phosphorylation may play an important role in mediating the effects of anti-cancer drug in the induction of apoptosis [25]. We report here that, upon the anti-cancer drug oxaliplatin treatment, p68 RNA helicase becomes threonine phosphorylated in colon cancer HCT116 cells. Oxaliplatin treatment activates p38 MAP kinase in the cells, which subsequently phosphorylates p68 at T564 and/or T446. Our results demonstrate that the phosphorylation of p68 at T564 and/or T446 is critically important for the apoptosis induction by the drug. Our studies reveal a very important molecular factor that mediates the effects of anti-cancer drug in apoptosis induction and may suggest a potential therapeutic strategy for cancer treatment.

## Results

### Oxaliplatin treatment of colon cancer cells induced p68 threonine phosphorylation

**Figure 1:**
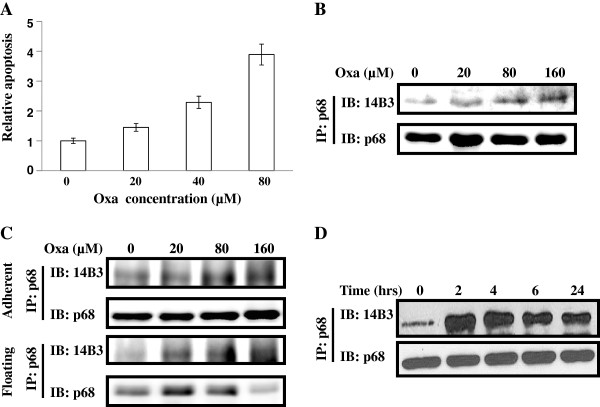
**Threonine phosphorylation of p68 in HCT116 cells under oxaliplatin (Oxa) treatment. **(**A**) Apoptosis of HCT116 cells under the treatment of different concentrations of oxaliplatin is measured by a commercial apoptosis kit and presented as Relative apoptosis by defining the apoptosis of the cells without oxaliplatin treatment as 1. Error bars represent standard deviations of four independent experiments. (**B**), (**C**), and (**D**) Threonine phosphorylations of p68 in HCT116 cells that are treated with different concentrations of oxaliplatin (**B**) and (**C**) or 20 μM of oxaliplatin for different times (**D**) are analyzed by immunobloting the p68 that is immunoiprecipitated (IP:p68) from cell lysates using antibody against phorsphor-threonine (IB:14B3). Immunoblot of p68 (IB:p68) in immunoprecipitates indicate the amounts of p68 were precipitated down. In (**C**), the analyses are carried out with the cells that are already floating (Floating) and the cells that are still adherent (Adherent) under the treatments.

We previously reported that tyrosine phosphorylation of p68 correlates with tumor progression [25]. In studying the effects of several anti-cancer drugs and p68 phosphorylation status, we noted that significantly higher levels of p68 threonine phosphorylation were observed in the colon cancer cells following the treatment with several anti-cancer drugs [25]. We asked whether the phosphorylation of p68 at threonine plays a role in mediating the anti-cancer drug effects. We used oxaliplatin that is commonly used in the treatment of colon cancer patients. Massive cell death of HCT116 cells was induced upon the treatment with the drug at different dosages (Figure 1A). We then examined the threonine phosphorylation of p68 by the procedure similar to that described in our previous reports [25,27]. It was clear that oxaliplatin treatment dramatically increased p68 phosphorylation at threonine (Figure 1B). This phosphorylation increase was observed with both the floating cells and the cells that were still attached to the culture plate under the drug treatment (Figure 1C). We subsequently tested the timing of the p68 threonine phosphorylation upon oxaliplatin treatment. It was evident that the p68 threonine phosphorylation reached a peak around 4 – 6 hours post drug treatment at the most commonly used drug concentration of 20 μM. The phosphorylation decreased thereafter (Figure 1D).

### P68 is phosphorylated by p38 MAP kinase at T564 and T446 upon the drug treatment

**Figure 2:**
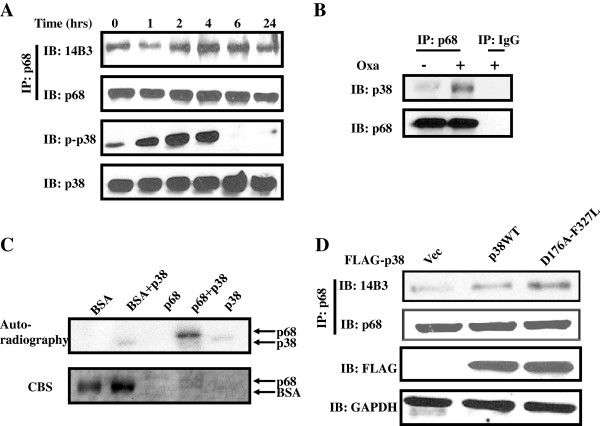
**MAPKPhosphorylation of p68 by p38 MAPK. **(**A**) Threonine phosphorylations of p68 in HCT116 cells that are treated with 20 μM of oxaliplatin for different times are analyzed by immunobloting the p68 that are immunoiprecipitated (IP:p68) from cell lysates using antibody against phorsphor-threonine (IB:14B3). Phosphorylation of p38 MAPK under the same treatment is analyzed by immunoblot of cell lysates using antibody against the phosphorylated p38. Immunoblot of p68 (IB:p68) in the immunoprecipitates indicate the amounts of p68 that are precipitated. Immunoblot of p38 in the cell lysate (IB:p38) indicate the cellular levels of p38, as a loading control. (**B**) Co-immunoprecipitation of p38 and p68 in the cell extracts of HCT116 cells with/without oxaliplatin treatment (Oxa, +/− 20 μM) was analyzed by immunoblot of p68 immunoprecipitates (IP:p68) using antibody against p38 (IB:p38). Immunoblot of p68 (IB:p68) in the immunoprecipitates indicate the amounts of p68 that are precipitated. IP:IgG is the immunoprecipitation using rabbit IgG, serving as a negative control IP. (**C**) Phosphorylation of recombinant His-p68 or BSA, as a control, by recombinant p38 in the presence of [γ-^32^P]-ATP is revealed by autoradiography. The amounts of proteins used in the phosphorylation reactions are shown by coomasie blue stains (CBS). (**D**) Phosphorylation of p68 by exogenous expression of Flag-tagged p38 MAPK, wild type and constitutively active mutant D176A-F327L, in HCT116 cells are analyzed by immunoblotting the p68 that are immunoiprecipitated (IP:p68) from cell lysates using antibody against phorsphor-threonine (IB:14B3). Immunoblot of p68 (IB:p68) in immunoprecipitates indicate the amounts of p68 that are precipitated. Immunoblot of Flag-tag (IB:FLAG) indicate the exogenous p38 levels. Immunoblot of GAPDH (IB:GAPDH) is a loading control.

We next sought to investigate the protein kinase that phosphorylates p68 in response to the anti-cancer drug treatment. It is well known that p38 MAP kinase is strongly activated in colon cancer cells upon the treatment by oxaliplatin. Activation of p38 MAP kinase is critical for the induction of apoptosis of the cancer cells [11,28]. To investigate the relation of p68 threonine phosphorylation and p38 MAP kinase activation, we simultaneously probed p68 phosphorylation and phosphorylation of p38 MAP kinase at the TGY motif using a commercially available antibody against the phosphorylated/activated p38 in HCT116 cells. It was clear that p38 MAP kinase was activated upon the oxaliplatin treatment. Interestingly, the timing of p38 MAP kinase activation/phosphorylation correlated very well with the p68 phosphorylation at threonine upon the oxaliplatin treatment (Figure 2A), indicating a possibility that p68 is a target of p38 MAP kinase during the induction of cell apoptosis by the anti-cancer drug. To test whether p68 is indeed the target of p38 MAP kinase, we examined the interaction of p68 and p38 by co-immunoprecipitation. HCT116 cells expressing endogenous p68 and p38 were treated by oxaliplatin. P68 was immunoprecipitated from the cell extracts prepared from the treated cells using anti-p68 antibody. It was evident that p38 coimmunoprecipitated with p68 (Figure 2B). We then further verified the phosphorylation of p68 by p38 MAP kinase by carrying out an *in vitro* phosphorylation using recombinant p68 and p38 MAP kinase. It was clear that the recombinant p68 was phosphorylated by the recombinant p38. As a control, BSA was not phosphorylated by the recombinant MAP kinase (Figure 2C). To further confirm that p38 indeed phosphorylated p68 at threonine residue, we used a constitutively activated p38 mutant D176A-F327L. D176A-F327L was expressed in HCT cells. Phosphorylation of p68 at threonine residue(s) in cells was examined by the immunoprecipitation and immunoblot procedures. Apparently, phosphorylation of p68 at threonine was dramatically increased upon the p38 mutant expression (Figure 2D). We concluded from our studies that p68 is phosphorylated by p38 MAP kinase upon the apoptosis induction by anti-cancer drug treatment.

**Figure 3:**
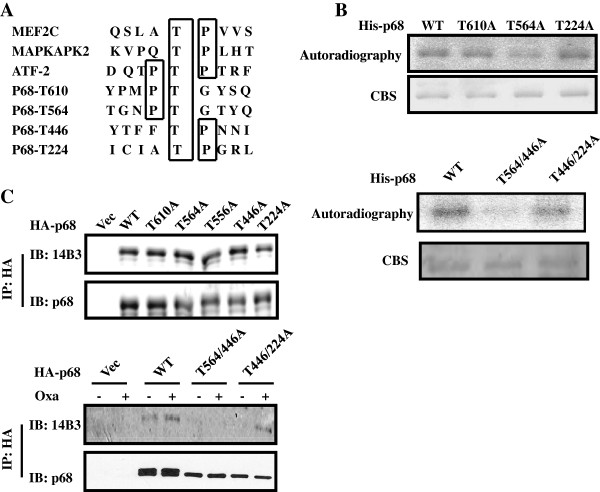
**Phosphorylation site(s) of p68 by p38 MAPK. **(**A**) Prediction of potential p38 MAPK phosphorylation site(s) in the p68 reading frame and compared to the consensus p38 MAPK phosphorylation sites of several authentic p38 MAPK substrates by a web-based phosphorylation site prediction program NetPhos 2.0, (**B**) Phosphorylation of recombinant His-p68 and mutants with single site mutation (Upper) and double site mutations (Lower) by recombinant p38 in the presence of [γ-^32^P]-ATP is revealed by autoradiography. The amounts of proteins used in the phosphorylation reactions are shown by coomasie blue stains (CBS). (**C**) Phosphorylation of exogenously expressed HA-p68s, wild type (WT) and mutants (Single site mutation, Upper, and Double site mutations, Lower), in HCT116 cells with/without oxaliplatin treatment (Oxa, +/−) are analyzed by immunoblotting the p68 that is immunoiprecipitated (IP:p68) from cell lysates using antibody against phorsphor-theronine (IB:14B3). Immunoblot of p68 (IB:p68) in immunoprecipitates indicate the amounts of p68 that are precipitated.

We next determined the potential p68 phosphorylation sites by p38 MAP kinase. We carried out a phosphorylation site search using a web-based program. The consensus phosphorylation site search indicated several potential S/T phosphorylation sites (Figure 3A). Based on the phosphorylation site prediction, we made several mutants that carried mutation at the predicted phosphorylation sites (Figure 3A). *In vitro* phosphorylation reaction with the generated mutants using the recombinant p38 indicated that there was a significant decrease in p68 phosphorylation with the mutant T564A, while there was almost no change with other mutants (Figure 3B, Upper panel), indicating that T564 is a potential site. To verify whether the T564 is the phosphorylation site, the T564A mutant or other mutants were expressed in HCT116 cells. After the cells were treated with oxaliplatin, phosphorylation of the p68 mutant at threonine was examined. Surprisingly, there was no change in p68 threonine phosphorylation with wild type and any mutant (Figure 3C Upper panel). One possible explanation is that p68 may have additional phosphorylation sites by p38 MAP kinase. It is well established that p38 MAP kinase often phosphorylates multiple sites in its targets [29,30]. To test this possibility, we created two p68 double mutants, T564/446A and T446/224A. The *in vitro* phosphorylation was carried out with these two mutants. It was clear that phosphorylation of T564/446A by p38 MAP kinase was almost abolished, while the phosphorylation of T446/224A had very minor reduction (Figure 3B Lower panel). The *in vitro* phosphorylation results suggested that it is likely that the T564 and T446 of p68 are the phosphorylation sites by p38. To verify whether indeed the T564 and T446 are the phosphorylation sites, HA-tagged p68 wt, T564/446A, and T446/T224A were expressed in HCT116 cells. The cells were treated by oxaliplatin. Phosphorylations of the HA-tagged p68 wt and the mutants were examined. Clearly, phosphorylation of T446/T224A experienced a minor decrease, while phosphorylation of T564/446A was almost abolished (Figure 3C Lower panel). The results strongly argued that p38 phosphorylated p68 at T564 and T446 upon the apoptosis induction by anti-cancer drug.

### Phosphorylation of p68 at threonine mediates the effects of oxaliplatin in the induction of apoptosis

**Figure 4:**
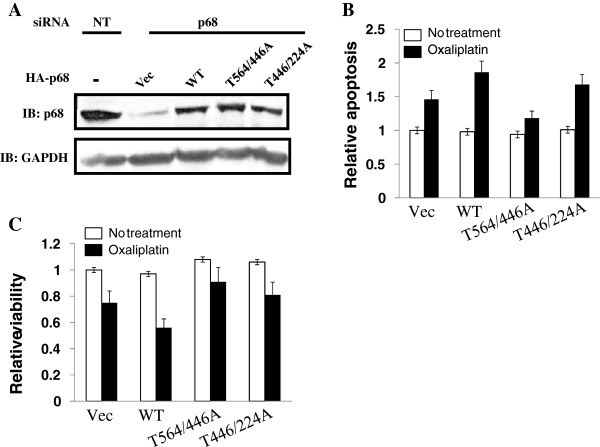
**Effects of p68 phosphorylation on cell apoptosis. **(**A**) HCT116 cells are treated by non-targeting siRNA (NT) or siRNA target p68 (p68). HA-p68, wild type and indicated mutants, are expressed in the p68 knockdown cells. Immunoblot of p68 (IB:p68) indicates the cellular levels of p68 (both endogenous and exogenous). Immunoblot of GAPDH (IB:GAPDH) is a loading control. (**B**) and (**C**). Cell apoptosis (**B**) and viability (**C**) of the cells in (**A**) that are treated or untreated with oxaliplatin are measured by a commercial apoptosis kit (**B**) or MTT assay (**C**). The cell apoptosis and viability are presented as relative apoptosis and relative viability by defining the apoptosis and viability of the vector expressing cells without oxaliplatin treatment as 1. Error bars represent standard deviations of four independent experiments.

We next investigated whether the p68 threonine phosphorylation by p38 plays a role in mediating the effects of the anti-cancer drug. To this end, the endogenous p68 was knocked down in HCT116 cells. HA-tagged at p68 or T564/446A was expressed in the p68 knockdown cells (Figure 4A). The cells were then subsequently treated by oxaliplatin at a concentration of 10 μM. Cell apoptosis was measured using a commercially available apoptosis assay kit (caspase-3 assay). There were no significant changes in cell apoptosis without oxaliplatin treatment. However, oxaliplatin induced apoptosis was dramatically reduced with the T564/446A expressing cells. This effect was not observed with the wt p68 expressing cells (Figure 4B). Similar results were observed with MTT assays (Figure 4C). Thus, we conclude that phosphorylation of p68 at T564/446 by p38, at least partially, mediates the effects of oxaliplatin on apoptosis induction.

## Discussion

In this report, we describe that p68 RNA helicase is phosphorylated at T564 and/or T446 in colon cancer HCT116 cells upon the anti-cancer drug oxaliplatin treatment. The protein is phosphorylated by p38 MAP kinase upon the drug treatment. The phosphorylation(s) of p68 contributes to the effects of apoptosis induction by the drug. Our results echo our previous report that the loss of p68 threonine phosphorylation correlates with cancer cell TRAIL resistance [27]. Thus, phosphorylation of p68 at T564 and T446 may represent a common molecular mechanism that acts in multiple pathways of apoptosis induction.

It is well established that activation of p38 MAP kinase is a common pathway for multiple apoptosis inducers, including a number of anti-cancer drugs with different molecular mechanisms [7,13,31], oxidative stresses [8], cells damaged by UV light [32]. However, the downstream targets that mediate the effects are not very clear. In fact, only a few substrates of p38 MAP kinase have been identified that have a role in cell apoptosis induction, including p53 [17] and HSP27 [33,34], and the mechanism by which phosphorylation of these substrates mediates the effects of apoptosis induction is not fully understood.

How the phosphorylation of p68 at T564 and T446 mediates cell apoptosis is an open question. One plausible explanation is that the phosphorylated p68 may change p68 interacting partners in the cells, which allows p68 to target a particular apoptosis mediating protein or complex. The consequence for the p68 targeting is activation of apoptotic function of the targeted protein or complex. It is known that a number of DEAD box RNA helicases associate with apoptosis induction protein or complex. For example DDX42 modulates the apoptotic function of ASPP2 by direct interaction with it [35]. Interaction between GABA receptor associated protein (GABARAP) and DDX47 is required for induction of neuronal cell apoptosis [36]. However, it is not known how this DEAD box RNA helicase regulates the apoptotic process. In this regard, it will be interesting to probe whether phosphorylation of p68 at T564 and/or T446 mediates the association of the protein with a particular apoptosis inducing complex to activate the complex. Among these potential interacting partners, p53 and p68 interaction is of particular intriguing, p68 was shown to interact with p53 to mediate the effects of p53 downstream targets. It is also well established that oxaliplatin induces cell apoptosis via activation of p38, which subsequently phosphorylates p53 in human colorectal cancer cells [37,38]. Thus, role of existence of p53 function in apoptosis induction related to the p68 Thr phosphorylation is an interesting potential.

## Methods

### Cell culture and antibodies

Human HCT116 cells were obtained from ATCC (Manassas, VA, USA) and were cultured according to vendor’s instruction. Antibodies against p68 were raised against bacterially expressed His-tagged C-terminal domain (a.a.437-614) of human p68 (Invitrogen, Carlsbad, CA, USA, Auburn University Hybridoma Facility). Antibodies against β-actin, phosphor-theronine (14B3), p38 and phosphor-p38, HA-tag, Flag-tag, and GAPDH were purchased from Santa Cruz, BD Bioscience, and Roche Applied Science respectively.

### Drug treatment, DNA constructs, transfections, and siRNA interference

Oxaliplatin was purchased from Sigma and dissolved in water to prepare a stock solution of 2.5mM. The stock solution was stored at −20°C and diluted with medium to prepare working concentrations. The cDNA of p68 ORF was subcloned into pHM6 vector (Roche) at HindIII site to get HA-tagged p68 expression vector. The various p68 single and double threonine mutants (threonine replaced by alanine) were generated by Quick-Change site-directed mutagenesis kit (Stratagene) and the mutations were confirmed by DNA sequencing. P38α cDNA (Origene) was subcloned into p3XFLAG-myc-CMV™-24 Expression Vector (Sigma). A number of mutations to get the constitutively active form of p38 were done using the reference [39]. All DNA transfections were performed using fugene HD (Roche) and lipofectamine 2000 (Invitrogen) while siRNA transfections were done with lipofectamine RNAimax (Invitrogen). The duplex siRNA against p68 was purchased from Dharmacon and the sequence was as follows: siRNA oligonucleotides against p68 (sense: GCAAGUAGCUGCUGAAUAUUU; antisense: AUAUUCAGCAGCUACUUGCUU). Cells were transfected with the indicated plasmids 24 hrs after p68 siRNA knockdown and further treated with the drug for 24 hrs. The cells were then harvested for nuclear extract preparation using a kit from Active motif.

### Protein expression and purification

The procedure used to express and purify p68 is similar to the procedure reported previously. P68 ORF was cloned and various mutants were cloned into expression vector pET-30a+ using the restriction sites BamHI/HindIII and transformed into *E.coli* BL21-CodonPlus bacteria (Stratagene) were used to express protein. The bacteria were subcultured in fresh st. LB broth till OD reached between 0.5 to 0.8 units at 600nm and then subsequently induced with 0.5mM IPTG for 18 hrs at 16°C. The cells were harvested, washed with 1X PBS buffer, pelleted and stored at −80°C. The cells were then disrupted by one freeze-thaw cycle at −80°C, resuspended in lysis buffer (50mM Tris–HCl pH 8.0, 300mM NaCl, 1mM DTT, 10mM PMSF, 10% glycerol) and subjected to lysozyme (0.5mg/ml) digestion. DTT and PMSF were also added at 1mM final concentration. The cells were further subjected to ultrasonication and pelleted. After centrifugation, the expressed protein was found to be precipitated in the bacterial inclusion bodies (IB). The IB were dissolved in denaturing buffer containing 8M urea, 50mM Tris–HCl pH 8.0, 250mM NaCl and 0.2% Triton-100. The lysate was passed through Ni-NTA column for purification of recombinant protein by affinity separation and the column was washed with the denaturing wash buffer (8M urea, 50mM Tris–HCl pH 8.0, 250mM NaCl, 0.2% Triton-100 and 20mM imidazole pH 8.0). The protein was finally eluted with elution buffer containing 250mM Imidazole, 8M urea, 50mM Tris–HCl pH 8.0, 250mM NaCl, 0.2% Triton-100, 0.5mM DTT and 10% glycerol. The eluted protein solution was refolded using stepwise dialysis procedure (8M→ 6M→ 4M→ 2M→ 0M) to remove urea using refolding buffer (200mM arginine, 50mM Tris–HCl pH 8.0, 250mM NaCl, 0.2% Triton-100, 0.5mM DTT and 10% glycerol) and preserved in further 15 to 20% glycerol.

### *In vitro* kinase assay

In this assay, about 1μg of purified proteins were added to the reaction mixture consisting of 200-250ng of p38α/SAPK2a enzyme (Upstate cell signaling), 10μCi/μl of [γ-32P]ATP, Magnesium/ATP cocktail (75mM MgCl2 and 500μM ATP in 20mM MOPS, pH 7.2, 25mM β-glycerol phosphate, 5mM EGTA, 1mM sodium orthovanadate and 1mM dithiothreitol) and 5X reaction buffer (125 mM Tris–HCl, pH 7.5 and 0.1mM EGTA) in a total volume of 25μl. After incubation at 30°C for 30 mins, 5X loading buffer (Fermentas) was added to stop the reaction. The samples were then analyzed by running two 10% SDS-PAGE gels. One of the gels was stained with Coomassie blue staining solution and destained and the other gel was dried and subjected to autoradiography.

### Cell viability and apoptosis assay

Cell viability of HCT116 cells was measured using (3-[4,5-dimethylthiazol-2-yl]-2,5-diphenyl tetrazolium bromide) or MTT (Sigma). 4000 cells were seeded per well of 96 well plate 24 hrs before knockdown and transfection of p68 and the mutants. Subsequently, 24 hrs after transfection, the cells were treated with oxaliplatin for 24 hrs. Next day, reconstituted MTT reagent was added in an amount equal to 10% of the culture medium volume and the cells were incubated at 37°C for a further 4 hrs. The formazan crystals were dissolved by adding MTT Solubilization solution. Cell viability was measured spectrophotometrically by reading the absorbance at a wavelength of 570 nm.

Cells plated on 6 well plates were treated with oxaliplatin after p68 knockdown and transfections and the activity of caspase-3 was measured using caspase-3/CPP32 colorimetric assay kit (Biovision Research products). Briefly, after apoptosis induction, the cells were resuspended in Cell Lysis Buffer for 10 mins and centrifuged. 50 μg of proteins were diluted in Cell Lysis Buffer to which 2X Reaction buffer and 4 mM DEVD-pNA substrate were added and incubated at 37°C. The samples were read at 405 nm using a microtiter plate reader. The cells were treated with oxaliplatin at previously indicated concentration and time and apoptosis was measured using FITC Annexin V Apoptosis Detection Kit (BDbiosciences). The brief description of the procedure is as follows: The cells were washed twice with cold 1X PBS after treatment and resuspended in 1X Annexin Binding Buffer at a final concentration of 1 x 10^6^ cells/ml. To 1 x 10^5^ cells (100μl) in a 5ml FACS tube, 5μl of FITC Annexin V and 5 μl of PI were added, gently vortexed and incubated at room temperature for 15 mins in the dark. Finally, 400μl of 1X Annexin Binding Buffer was added and the samples were analyzed by flow cytometry within 1hr.

## Competing interests

The authors declare that they have no competing interests.

## Authors’ contribution

Heena Dey carried out all the experiments and analysis of experimental data. She also contributed partially to experimental designed and to the preparation of the manuscript. ZR Liu is responsible for experimental design and the preparation of the manuscript. All authors read and approved the final manuscript.
